# Punishment-based decision making

**DOI:** 10.3389/fnins.2013.00236

**Published:** 2013-12-10

**Authors:** Jean-Claude Dreher, Ben Seymour, Philippe N. Tobler

**Affiliations:** ^1^Reward and Decision Making Group, Centre de Neurosciences Cognitives, Centre National de la Recherche ScientifiqueLyon, France; ^2^Center for Information and Neural Networks, National Institute for Information and Communications TechnologyOsaka, Japan; ^3^Laboratory for Social and Neural Systems Research, Department of Economics, University of ZurichZurich, Switzerland

**Keywords:** punishment, reward, decision making, dopamine, serotonin

This Research Topic covers issues in psychology, behavioral economics, and cognitive neuroscience investigating the neural structures and mechanisms underlying approach, and avoidance behavior in the face of rewards and punishments. The objective is to understand the nature of critical differences and asymmetries between the ways that appetitive and aversive outcomes are processed by the brain. A number of topics are covered, such as the development of economic models integrating costs and benefits into a single value, neuroimaging approaches of appetitive and aversive conditioning, reward-punishment interactions, pain and defensive behavior, the role of dopamine neurons in aversive conditioning, and the interactions between serotonin and dopamine in punishment, pain, and aversion. The neural bases of reward-punishment interactions are of great interest to a broad readership because of the fundamental role of dopamine and serotonin in a number of motivational and decision processes, and because of their theoretical and clinical implications for understanding dysfunctions of these two systems. Findings in this research field are also important to basic neuroscientists interested in the computational processes of pain and aversive learning and cognitive psychologists working on conditioning/reinforcement. Punishment-based decision making and reward processing cover a wide range of topics and levels of analysis, from basic neural mechanisms and computational models of appetitive and aversive conditioning, to the system neuroscience level. The contributions to this Frontiers Research Topic in Decision Neuroscience are forward-looking assessments of the current and future issues faced by researchers.

## Research articles

Porcelli et al. (2012) investigate how stress influences reward and punishment processing neural circuitry. They report results from a new fMRI study where participants were exposed to acute stress or a no stress control procedure and subsequently performed a fMRI paradigm where they received monetary rewards and punishments. Acute stress group participants' dorsal striatum and orbitofrontal cortex response demonstrated decreased sensitivity to monetary outcomes and a lack of differential activity. The reported findings provide insights into how neural circuits may process rewards and punishments associated with simple decisions under acutely stressful conditions.

In a second study, Singh and Khan (2012) studied the effect of reward and punishment sensitivity on long-term advantageous decisions in two variants of the Iowa gambling task (IGT). The results indicate that foresight in IGT decision making is sensitive to reward and punishment frames in an asymmetric manner. Moreover, variant, order, and instruction types had an effect on long-term decision making in the IGT.

In the third article, Rigoli et al. (2012) studied how aversive Pavlovian responses affect instrumental motor performance. Based on animal studies which have demonstrated that Pavlovian mechanisms can have maladaptive effects on instrumental performance, the authors report that Pavlovian responses influenced performance, and can also have maladaptive effects in humans. In particular, Pavlovian responses either impaired or increased performance depending on variables such as threat distance, task controllability, punishment history, amount of training and explicit punishment expectancy. Overall, these findings help to elucidate the mechanisms underlying the interaction between Pavlovian and instrumental-performance.

## Review articles

Barberini et al. (2012) focus on reviewing neural signals during and after learning in the amygdala and orbitofrontal cortex, two brain areas that process appetitive and aversive stimuli. They reveal a dynamic relationship between appetitive and aversive circuits which shifts as a function of learning. Furthermore, although appetitive and aversive circuits may often drive opposite behaviors, these circuits can also drive similar behaviors, such as enhanced arousal or attention. These data highlight the existing challenges to pinpoint how appetitive and aversive neural circuits interact to produce a range of behaviors.

In a review article, Kobayashi (2012) extends the previous mini-review in several ways. He presents the medial pain system, including the amygdala, periaqueductal gray (PAG), and anterior cingulate cortex (ACC), that signals pain and negative value. He reviews behavioral and physiological studies on the aversive system and proposes a conceptual framework for understanding the neural organization of the aversive avoidance system. According to this framework, it is possible to distinguish between a medial system including the amygdala-PAG-orbitofrontal cortex (OFC), and ACC, serving as a predictor and evaluator of behavioral outcomes, and a lateral system, which includes the lateral prefontal cortex and receives multimodal sensory inputs.

Wiech and Tracey (2013) review the relationship between pain and motivational states, providing an overview on behavioral and neuroimaging studies investigating motivational aspects of pain. They highlight insights into the modulation of pain through fear and social factors, summarize findings on the role of pain in fear conditioning, avoidance learning and goal conflicts and discuss evidence on pain-related cognitive interference and motivational aspects of pain relief.

In a mini review, Ilango et al. (2012) examine the role of dopamine in response to aversive stimuli. The authors review data from electrophysiology, microdialysis and voltammetry describing dopamine changes in response to aversive stimuli and fearful events. For example, they show that dopamine neurons respond to aversive stimuli primarily with inhibition. They also describe the role of dopamine manipulations on signaled avoidance learning, which consists of learning the significance of a warning cue through Pavlovian associations and the execution of an instrumental avoidance response. They present a framework to understand the involvement of reward circuit in punishment based decisions.

In another paper, McCutcheon et al. (2012) review data indicating that Nucleus Accumbens (NAc) shell dopamine responses match the hedonic value of stimuli. They also present new data showing that oral infusion of sucrose suppresses instead of enhances NAc shell dopamine if the sucrose has been rendered aversive through previous pairing with malaise-inducing injection of lithium chloride. Sucrose infusions led to a suppression of dopamine with a similar magnitude and time course to intra-oral infusions of quinine solution. The results are discussed in the context of regional differences in dopamine signaling in the NAc.

Finally, Talmi and Pine (2012) review behavioral economic literature and describe models integrating costs and benefits into a single subjective value. They propose ways to assess these models beyond goodness of fit, such as how to model decisions between costs when reward is not on offer and whether these models predict changes in reward sensitivity when costs are added to outcomes. They also provide a selective review of relevant neurobiological work from a computational perspective, focusing on neuroimaging studies focusing on valuation mechanisms.

We anticipate that while some readers may read this Frontiers Research Topic from the first to the last chapter, other readers may read only one or more chapters at a time, and not necessarily in the order presented in this e-book. This is why we encouraged an organization of this volume whereby each chapter can stand alone, while making references to others and minimizing redundancies across the e-book.

Given the consistent acceleration of advances in the different approaches described in this Research Topic, we hope that you will enjoy these new stages of an exciting era in neuroscience research on the interactions between appetitive and aversive systems.
